# The safety and efficacy of endoscopic ultrasound-guided portal pressure gradient measurement with concomitant endoscopic ultrasound-guided liver biopsy: a systematic review

**DOI:** 10.3389/fgstr.2023.1209539

**Published:** 2023-11-17

**Authors:** Alexander Malik, Muhammad Nadeem Yousaf, Ghassan M. Hammoud

**Affiliations:** ^1^ Department of Medicine, Summa Health System, Northeast Ohio Medical University, Akron, OH, United States; ^2^ Department of Medicine, Division of Gastroenterology and Hepatology, University of Missouri, Columbia, MO, United States

**Keywords:** endoscopic ultrasound, portal hypertension, portal pressure gradient, liver biopsy, cirrhosis, fine needle aspiration, chronic liver disease

## Abstract

**Introduction:**

Portal hypertension (PH) is a complication of advanced liver disease. Traditionally, PH has been quantified using hepatic venous pressure gradient (HVPG) through an indirect transjugular approach requiring ionizing radiation exposure. Endoscopic ultrasound-guided porto-systemic pressure gradient (EUS-PPG) measurement is an emerging alternative, minimally invasive technique that provides direct portal pressure measurement. The aim of this systematic review is to evaluate the safety and efficacy of EUS-PPG measurement and concomitant EUS-guided liver biopsy (EUS-LB) in patients with chronic liver disease.

**Methods:**

The preferred reporting items for systematic reviews and meta-analyses method was used. A PubMed, Medline, Web of Science, Google Scholar, and CINAHL search for terms “endoscopic ultrasound,” “EUS,” and “portal pressure gradient” was used to identify qualifying studies. Eligible studies included those which were published before 2022, reporting outcomes of EUS-PPG measurement, simultaneous EUS-LB if applicable, and adverse events rate. Risk of bias was assessed by Egger’s test. Results were synthesized using I^2^ to test heterogeneity.

**Results:**

Four published studies including 147 patients met inclusion criteria, with mean age 59.6 years, 59% male. Indications for EUS-PPG measurement were history of chronic liver disease or suspected cirrhosis, viral hepatitis, alcohol associated liver disease, hepatic sinusoidal obstruction or Budd Chiari syndrome. The pooled technical success rate of EUS-PPG measurements was 98.61% with 95% confidence interval of 95.20% - 99.82%. A 25-gauge needle was used in 92% (135/147) of patients. EUS-PPG measurement was performed through a transgastric approach in all 147 (100%) patients using a compact manometer with pressure transducer and non-compressible tubing. The mean PPG was 10.07 (range 6.44 – 13.70) mmHg. Ninety-five patients underwent simultaneous EUS-LB using 19G needle with wet suction technique. Technical success rate of EUS-LB was 100% and specimen was adequate in 99% (94/95) patients to establish histological diagnosis. There were no major life-threatening complications of the EUS-PPG procedure. Predominant adverse events were abdominal pain 6.1% (9/147) and sore throat 5.4% (8/147).

**Conclusion:**

EUS-PPG measurement is safe and useful in providing an assessment of portal pressure in patients with chronic liver disease. Future studies are needed to evaluate whether there is consistent correlation between EUS-PPG measurements and histologic fibrosis stage by liver biopsy.

## Introduction

Portal hypertension (PH) is the result of elevated pressure within the portal venous system that may occur in the setting of advanced liver disease, right- sided congestive heart failure and thrombosis within the portal vascular system (1). Complications of PH contributing to significant morbidity and mortality in patients with advanced liver disease include bleeding from esophagogastric varices or portal hypertensive gastropathy, ascites, renal insufficiency, hepatic encephalopathy, and cirrhosis (1). Importantly, PH often develops prior to a histologic diagnosis of cirrhosis (2). Since direct access of the portal vein is not feasible, hepatic venous pressure gradient (HVPG) is an indirect method for measuring the portosystemic pressure gradient (PSG) and remains the gold standard method (3, 4). Based on the HVPG, clinically significant PH is defined as portal pressure >10mm Hg. HVPG is typically measured by an interventional radiologist using fluoroscopic guidance by a balloon-tipped catheter introduced into the hepatic vein via the internal jugular, antecubital or femoral veins (5, 6). HVPG is calculated as the difference between the free and wedge hepatic vein pressure (FHVP and WHVP, respectfully). Importantly, useful application of HVPG data is not possible in the setting of pre-hepatic or presinusoidal PH (7, 8). This traditional method provides an indirect measurement of the portal pressure gradient and requires exposure to ionizing radiation and intravenous contrast. Hence, there is a need for an alternative, minimally invasive method to measure direct portal pressure.

Endoscopic ultrasound-guided portal pressure gradient (EUS-PPG) measurement emerged as a minimally invasive technique of measuring direct portal pressure using a linear echoendoscope, 25-gauge fine needle aspiration (FNA) needle, and compact pressure manometer to access the portal and hepatic veins or IVC via a transgastric or transduodenal approach. The technique has been described previously by Huang et al. (9). The patient is placed in a supine position, and the manometer is set to zero prior to echoendoscope insertion by placing the manometer along the mid-axillary line at the level of the heart (9). Endoscopic ultrasound with Doppler study is used to identify either the middle or left hepatic vein. The hepatic vein is punctured with the 25-gauge FNA needle via the transgastric, transhepatic approach (10). After flushing approximately 1mL of normal saline and producing a transient rise in the pressure reading on the manometer, the pressure decreases to a steady level within 45 to 60 seconds and can be recorded as the free hepatic venous pressure (HVP) (9). The portal vein pressure (PVP) is obtained by first identifying the left portal vein using endoscopic ultrasound with Doppler study which is then punctured with the same 25-gauge FNA needle via a transduodenal (or transgastric), transhepatic approach. After flushing approximately 1mL of normal saline and producing a transient rise in the pressure reading on the manometer, the pressure decreases to a steady level within 45 to 60 seconds and can be recorded as the PVP. The PPG is then calculated based on the difference between PVP and HVP.

The EUS-PPG measurement technique has been demonstrated to be a safe and efficacious method for measuring PPG in the previous literature (2, 8, 9). Currently the EUS-PPG measurement technique is performed primarily at specialized centers by expert endosonographers. The purpose of this systematic review is to evaluate the feasibility and safety of the EUS-PPG measurement and concomitant EUS guided liver biopsy (EUS-LB). Additionally, this systemic review discusses clinical scenarios in which the EUS-PPG measurement is useful for patient care and potential areas for future investigation.

## Methods

**Figure 1:**
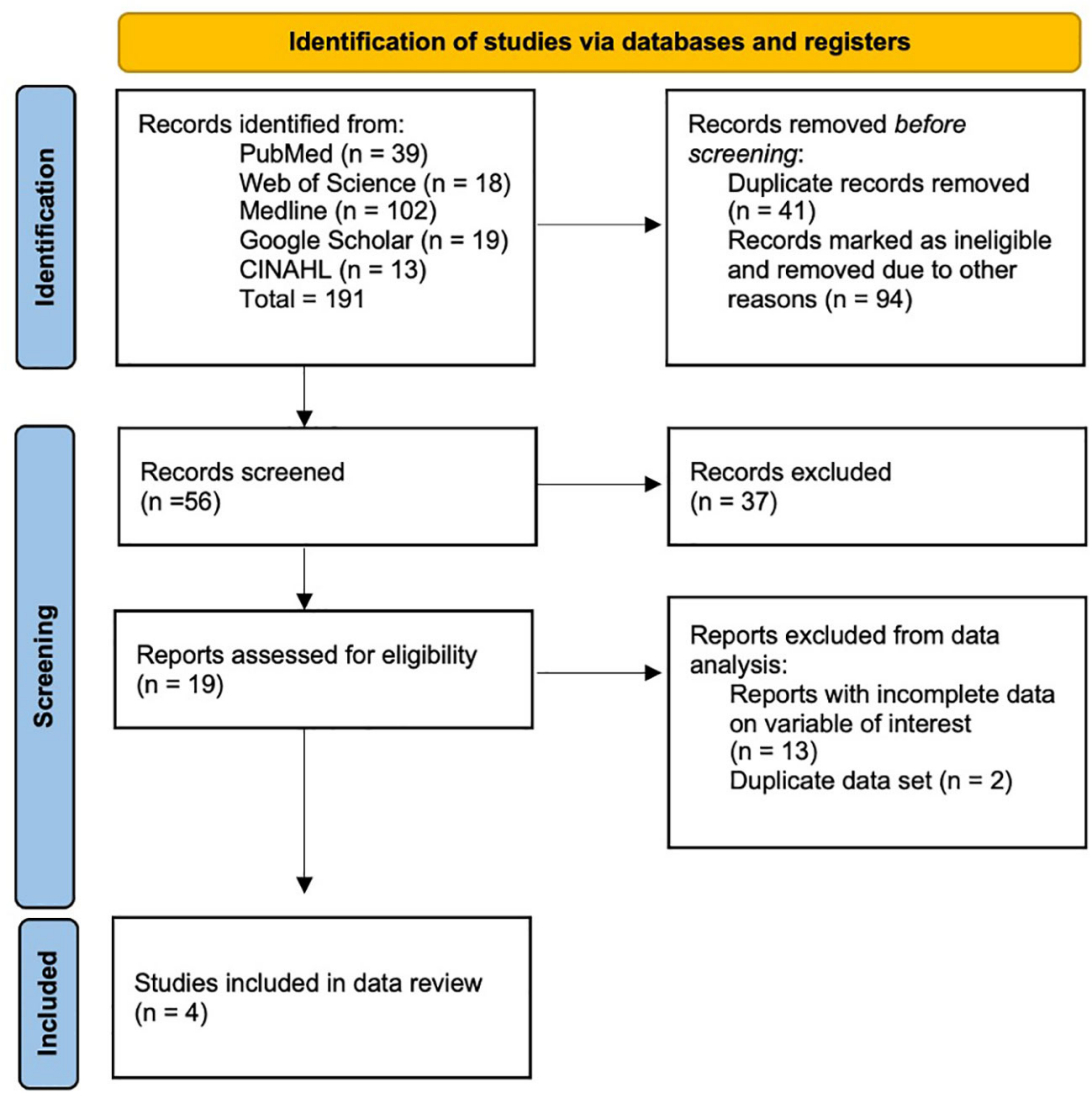
PRISMA flowchart showing study search strategy.

The preferred reporting items for systematic reviews and meta-analyses (PRIMSA) method was used. A comprehensive search on PubMed, Medline, Web of Science, Google Scholar, and CINAHL was made using terms “endoscopic ultrasound,” or “EUS,” and “portal pressure gradient” with Boolean operator, “and” to identify relevant studies (**Figure 1**). Eligible studies reporting the outcomes of technical success of the EUS-PPG measurement, the technical success of simultaneous liver biopsy (LB) were included with literature search as of October 2022. All abstracts without available manuscripts were excluded. A total of two independent reviewers screened each of the queried reports, without use of automation tools to determine eligibility. A single reviewer collected data from the eligible studies that was reviewed by another independent reviewer. For each study, the following data was collected:

I. Patient demographics (mean age, sex).
II. Clinical variables [indications for the EUS PPG measurement, Child-Turcotte-Pugh (CTP) score, status of esophageal/gastric varices, presence of portal hypertensive gastropathy (PHG)].
III. Procedure outcomes (needle used for EUS PPG, EUS-guided needle insertion approach, mean PPG measurement, presence of clinically significant PH (CSPH, defined as an HVPG ≥ 10 mmHg), technical success rate of EUS-PPG measurement, needle used for EUS-guided LB (EUS-LB), technical success rate of obtaining LB, specimen adequacy to establish diagnosis and staging of liver fibrosis.
IV. Adverse events rate.

For studies which did not report on one of the included variables, no assumptions were made, and the variables were simply omitted. Risk of bias was assessed by the two reviewers by examining the original studies. All data collected was tabulated using Microsoft Excel. The results were reported descriptive using mean (SD), median (IQR), range (min - max) or proportions depending on how the data was reported in the included studies. The data from multiple studies have been collated together using pooled proportion (for proportion) or generic inverse variance (for mean) method. Heterogeneity across the studies was quantified using the I^2^ statistic, and the I^2^ >50% indicated significant heterogeneity. The fixed-effect analytical model was used to pool the results of studies with acceptable or no heterogeneity. The random-effect model was used to analyze the results of studies with significant heterogeneity. Publication bias was assessed based on Egger’s test. A *p* value ≤0.05 was considered for statistical significance. All statistical analyses were performed with R software version 3.1.0.

## Results

In the initial search, 191 studies were found, out of which 56 were selected, and data was extracted from 4 studies. Two different manuscripts published by Huang et al. in 2017 and 2018, respectively, were analyzed. Both manuscripts utilized the same data set. Thus, for the purpose of preventing data overlap, the data sets were not double-counted in the data synthesis. Data from a manuscript published by Choi et al. in 2022 including 64 patients was excluded from the data synthesis to prevent data overlap, as a later manuscript published by the same author set included 83 patients from the same institution over a slightly different study duration.

### Individual studies characteristics

In 2017, Huang et al. published a prospective cohort pilot study which included 28 patients who underwent EUS-PPG measurement under either moderate sedation or general anesthesia at a single institution (9). Indications for the procedure included history of chronic liver disease or suspected cirrhosis. The technical success of the EUS-PPG measurement was 100% and the mean PPG measurement was 8.2 mmHg (range 1.5-19 mmHg). There were no reported complications of the procedure. Higher PPG levels were correlated with high clinical evidence of cirrhosis (Wilcoxon Rank Sum Test, *p=*0.005), and in those with esophageal and/or gastric varices (nominal *p=*0.0002), portal hypertensive gastropathy (PHG) (*p=*0.007) and thrombocytopenia (*p=*0.036), compared to those without advanced liver disease. Based on the logistic regression models utilized, when a patient had PPG ≥5 mmHg, the odds of high evidence of cirrhosis was 18.7 (95% CI, 2.97-180.66) times higher than a patient with a normal PPG (< 5 mmHg) measurement. Overall, the authors reported excellent correlation between PPG measurement and clinical evidence of cirrhosis.

In a pilot study, Zhang et al. included 12 patients with acute or subacute portal hypertension because of pyrrolizidine alkaloid-induced hepatic sinusoidal obstruction syndrome or Budd Chiari syndrome (11). There were nine patients with CTP score of 7-9 points and three with score of 10-15 points. Eleven of the 12 included patients underwent successful EUS-PPG measurement under moderate sedation. Following the EUS-PPG measurement, the 11 patients underwent HVPG measurement while conscious. HVPG measurement was inaccurate in one patient due to a small shunt and failed in two other patients due to Budd-Chiari syndrome (hepatic vein occlusion subtype). Thus, the final analysis compared nine patients who underwent successful transgastric, transhepatic EUS-PPG measurement and HVPG/transjugular PPG measurement. The mean EUS-PPG measurement was 18.07 ± 4.32 mmHg and the mean HVPG was 18.82 ± 3.43 mmHg. There were no reported adverse events attributed to either of these procedures. Overall, the authors reported a “high degree of consistency between EUS-PPG and the criterion standard test, HVPG” and strong correlation between PSG measurements and clinical manifestations of portal hypertension, including abdominal distention and ascites. The authors concluded that “EUS-PPG measurement is feasible, safe and effective” and it may be an alternative option for PSG measurement in patients for whom HVPG measurement is inaccurate or impossible.

In 2022, Hajifathalian et al. published the results of a pilot study including 24 patients with suspected chronic liver disease or cirrhosis who underwent simultaneous EUS-PPG measurement and EUS-LB using a 19G needle with wet suction technique under general anesthesia (12). The technical success of EUS-PPG measurement was 96%, with a mean PPG of 7.5 ± 4.32 mmHg. All 24 LB specimens were adequate for establishing histologic diagnosis and revealed advanced liver disease with fibrosis stage of 3 or greater in 10 patients (42%). There was one report of abdominal pain following the procedure. Overall, the authors demonstrated that simultaneous EUS-PPG measurement and EUS-LB are both feasible and safe. There was no statistically significant correlation between the fibrosis stage on histology and measured PPG, which was attributed by the authors to a type II sampling error, and low powered study due to small sample size. There was significant association between PPG and liver stiffness on transient elastography (*p*=0.011) and FIB-4 score (*p*=0.026).

In a retrospective study including 83 patients with history of chronic liver disease or suspected cirrhosis, Choi et al. described the correlation between EUS-PPG measurement and histological hepatic fibrosis (8). Data collection occurred from February 2014 to March 2020. EUS-PPG measurement was successful in all 83 patients, with mean PPG of 7.06 ± 6.09 mmHg. Seventy-one patients had a simultaneous EUS-LB performed using a 19-gauge needle during the procedure, with 70 of 71 biopsy specimens were adequate for histologic diagnosis. Both procedures were performed under general anesthesia. Adverse events from the procedure included mild abdominal pain and sore throat present in eight patients (9.6%). PPG was higher in patients with clinical features of cirrhosis (9.46 vs 3.61 mmHg, *p* < 0.0001), esophageal or gastric varices (13.88 vs 4.34 mmHg, *p* < 0.0001), and thrombocytopenia (9.25 vs 4.71 mmHg, *p* = 0.0022). A PPG > 5 mmHg predicted 13 times higher chance of cirrhosis compared with PPG < 5 mmHg (OR 13.15, 95% CI 4.56–44.86, *p* < 0.0001). PPG > 10 mmHg predicted 12 times higher chance of cirrhosis compared with PPG < 10 mmHg (OR 12, 95% CI 3.14–79.26, *p* = 0.0015). In a separate manuscript which included 64 patients from the same institution studied during the period of January 2014 to March 2020, the authors again assessed the primary outcome of association between EUS-PPG and presence of histologic liver fibrosis stage 3 or greater (2). EUS-PPG > 5 mmHg (vs. EUS-PPG < 5 mmHg) had significantly higher rate of fibrosis stage ≥3 on EUS-LB (78.6% vs. 27.6%, *p* = 0.02). Additionally, on multivariate analysis, EUS-PPG >5 mmHg was significantly associated with fibrosis stage ≥ 3 on EUS-LB (LR 27.0, 95% CI = 1.653–360.597, *p* = 0.004), independent from clinical cirrhosis, clinical poral hypertension, thrombocytopenia, splenomegaly, AST to platelet ratio index (APRI) score > 2, and FIB-4 score > 3.25. Overall, the authors concluded that EUS-PPG ≥5 mmHg is not only significantly and independently associated with stage 3–4 fibrosis, but it is also superior to several other commonly used variables and mathematic models to predict stage 3–4 fibrosis.

### Data synthesis

A total of 4 studies (2 retrospective and 2 prospective) involving 147 patients (56% males) with a mean ± standard deviation (SD) age of 59.6 ± 4.7 years having chronic liver disease, cirrhosis, or PH were included (**Table 1**). The median (range) Child Pugh Score was 8.5 (7-10) with 75% having CTP B and 25% CTP C in a study by Zhang et al.; while in study by Choi and colleagues 76% patients had CTP A, 18% CTP B and 6% CTP C disease. In a study by Hajifathalian et al. the mean ± SD FIB-4 score was 1.82 ± 1.8 with 50% having fibrosis stage 0-1, 21% having stage 2 or 3 and 29% having stage 4 based on Fibroscan and the mean Model for End-Stage Liver Disease with Sodium (MELD-Na) score was 8 among stage 3 or 4 fibrosis patients. The pooled (95% CI) proportion of patients for the presence of esophageal/gastric varices was 29.55% (22.41% - 37.51%) {Heterogeneity: I^2^ (95% CI): 10.46% (0.00% - 88.44%); *p*=0.3407; 4 studies; fixed effect model} with no evidence of publication bias (Egger’s test *p*=0.9311) *(* 8, 9, 11, 12). The approach used in all the studies for EUS-PPG was transgastric transhepatic using compact manometer with pressure transducer and non-compressible tubing catheter (8, 9, 11, 12). The proportion of patients with clinical evidence of PH on esophagogastroduodenoscopy (EGD) was 32.6% [48/147; 4 studies (8, 9, 11, 12)], portal hypertensive gastropathy on EGD was 34.2% [38/111; 2 studies (8, 9)], and clinically significant PH was 37.7% [23/61; 3 studies (8, 9, 12)].

**Table 1:** Variables available in analyzed studies.

Study	Year	Total patients	Male patients	Mean age (years)	Clinical evidence of PH*	Needle used for EUS-PPG procedure (gauge)	EUS-PPG Technical Success Rate (%)	Mean PPG, mmHg	Liver biopsy attempted (Y/N)?	Liver biopsy specimen adequacy for diagnosis	Adverse Events Related to Procedure
**Huang (** 9)	2017	28	18	63	11	25	28/28 (100)	8.2	N	NA	None
**Zhang (** 11)	2021	12	9	63	4	22	11/12 (91.70)	18.07	N	NA	None
**Choi (** 8)	2022	83	51	59.4	29	25	83/83 (100)	7.06	Y, in 71 patients	70/71	Mild abdominal pain and sore throat (8)
**Hajifathalian (** 12)	2022	24	5	53	4	25	23/24 (96)	7.5	Y, in 24 patients	24/24	Abdominal pain (1)
**Total**		147	83	59.6	48	25G: 135/147 (91.8%)	145/147 (98.6)	7.06 +/- 2.495	95 attempts	94/95 (98.9)	9

PH, portal hypertension; EUS-PPG, endoscopic ultrasound guided portal pressure gradient; PPG, portal pressure gradient; Y, yes; N, no; NA, not applicable.

*Defined as presence of esophageal and/or gastric varices or portal hypertensive gastropathy on endoscopy, ascites, or splenomegaly (≥12 cm on cross-sectional imaging) plus platelet count <150,000/mL3.

**Figure 2:**
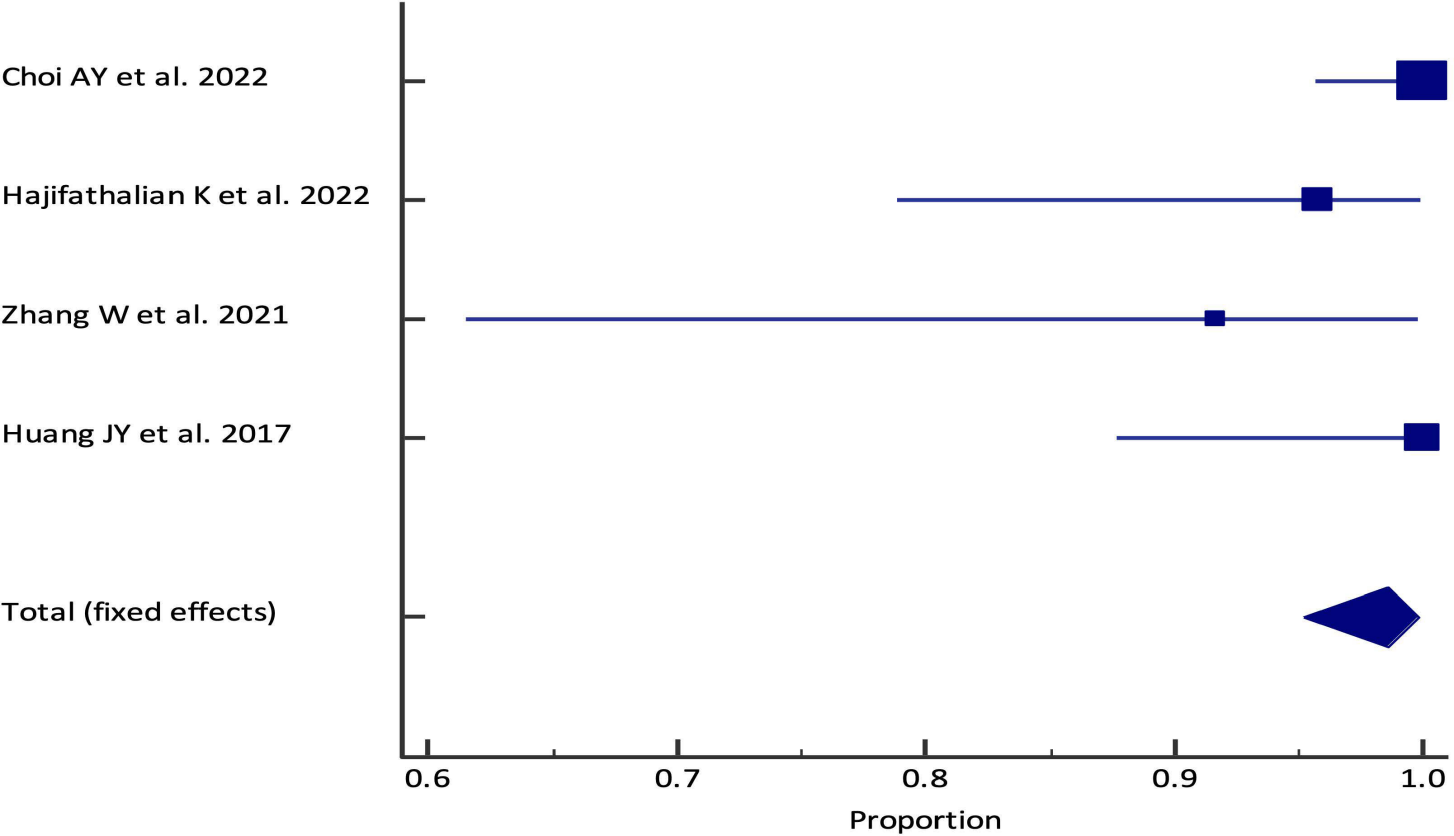
Forest plot of the pooled analysis for EUS-PPG measurements technical success rate among patients across different studies. The lower diamond in the graph represents the pooled estimate.

**Figure 3:**
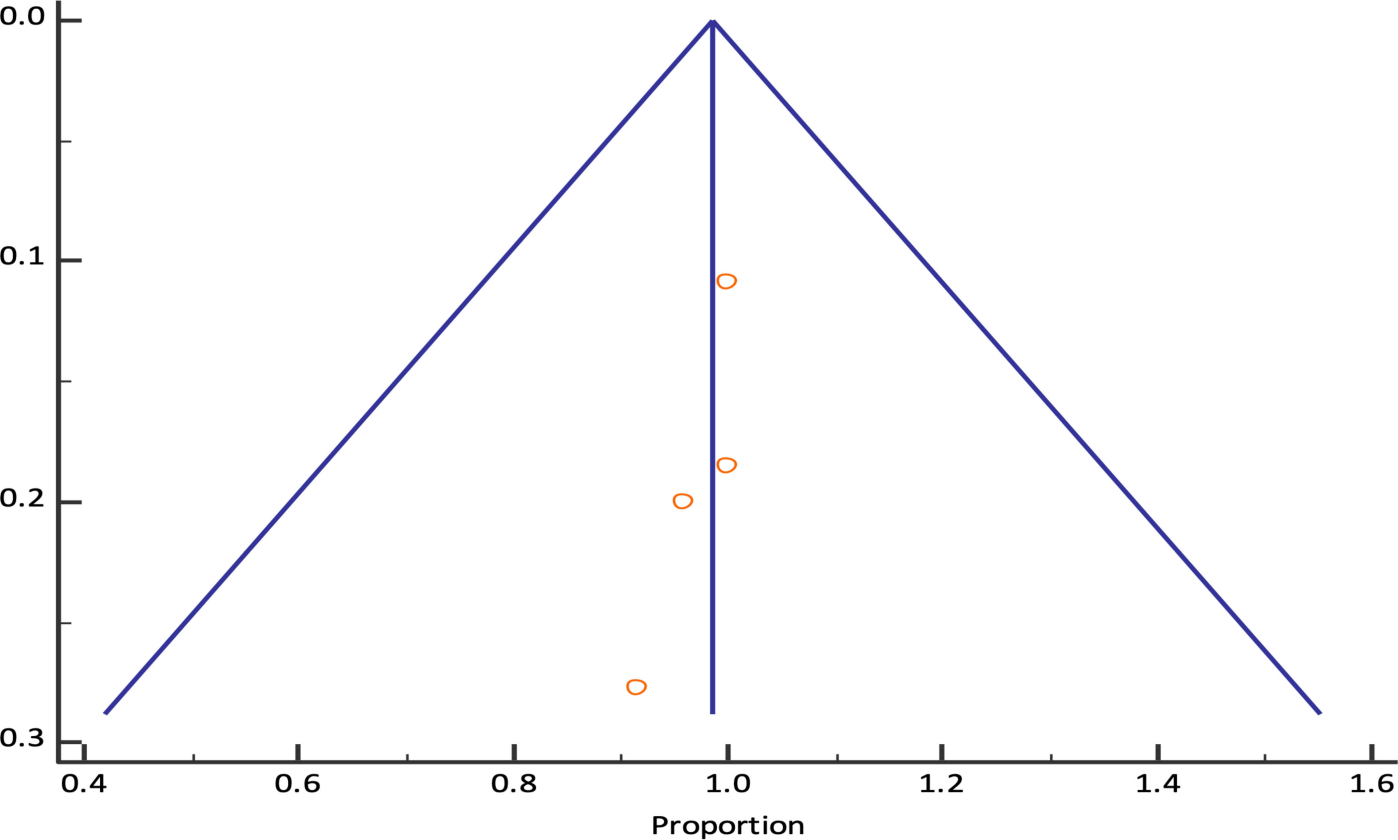
Funnel plot for EUS-PPG measurements technical success rate among patients across different studies.

**Figure 4:**
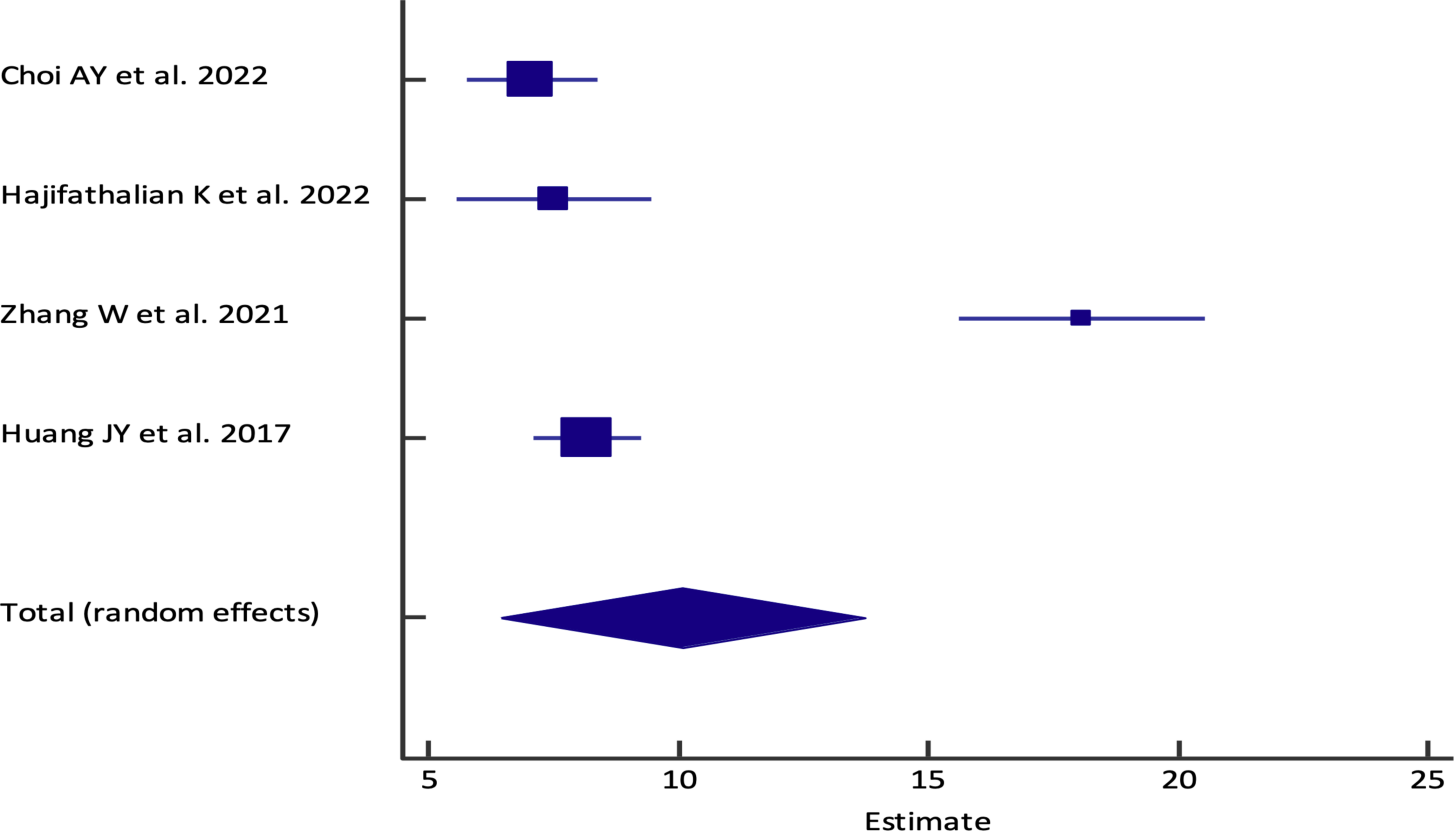
Forest plot of the pooled analysis for EUS-PPG among the patients across different studies. The lower diamond in the graph represents the pooled estimate.

**Figure 5:**
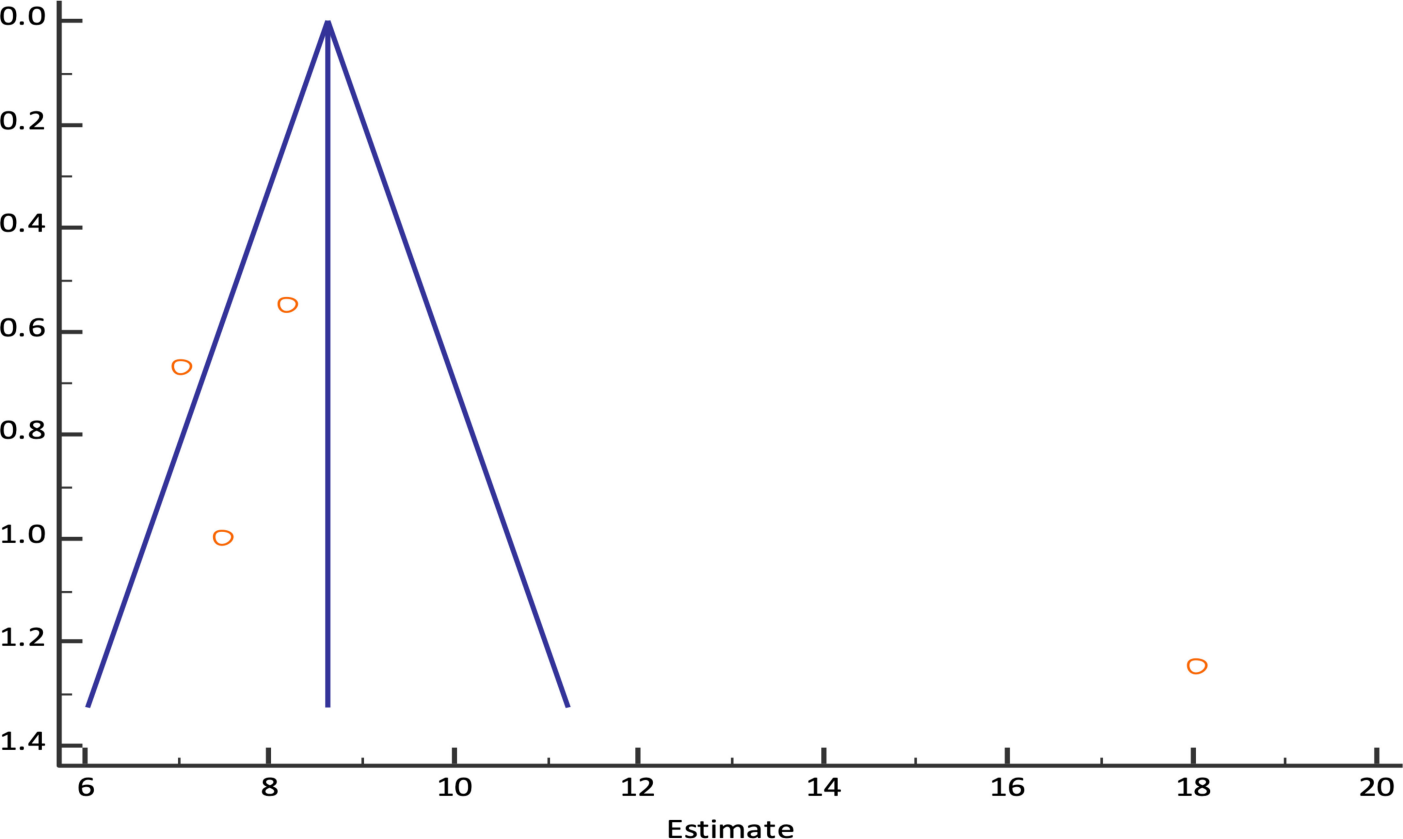
Funnel plot for EUS-PPG among the patients across different studies.

The pooled technical success rate was 98.61% (95% CI; 95.20% - 99.82%) {Heterogeneity: I^2^ (95% CI): 46.59% (0.00% - 82.26%); *p*=0.1318; 4 studies; fixed effect model (**Figure 2**)} with no evidence of publication bias (Egger’s test *p*=0.09) *(* 8, 9, 11, 12) (**Figure 3**). The pooled (95% CI) mean EUS-PPG among the patients across the included studies was observed to be 10.07 (6.44 - 13.70) mm Hg {Heterogeneity: I^2^ (95% CI): 95.34% (90.99% - 97.59%); *p*<0.0001; 4 studies; random effect model (**Figure 4**)} with no evidence of publication bias (Egger’s test *p*= 0.3132) (8, 9, 11, 12) (**Figure 5**). The mean ± SD hepatic venous pressure gradient was 17.36 ± 3.87 mm Hg based on two studies (9, 11). The procedure time was <60 minutes (two studies). The liver biopsy specimen was observed to be adequate and wet suction was the most common liver biopsy technique in almost 99% (94/95) of the patients across the two studies where EUS-LB was performed (8, 12).

### Adverse event

No adverse event was observed by Huang et al.; minor events such as abdominal pain 8.4% (9/107) and sore throat 7.5% (8/107) were observed in 2 studies (8, 12).

## Discussion

The prevalence of chronic liver disease is significant and as of 2020, 1.5 billion patients are estimated to have chronic liver disease worldwide (13). The presence or absence of CSPH is particularly important for determining whether a patient with cirrhosis progresses to develop complications that define decompensated cirrhosis, including ascites, hepatic encephalopathy, and variceal bleeding. That is, patients with cirrhosis can be divided into two stages, the first being those without CSPH and the second, those with CSPH who are at risk of decompensation (3). With the significant morbidity associated with complications of PH, methods of accurately measuring the PPG that simultaneously minimize patient complications are essential.

The studies included in this review demonstrated that the technical success rate of the EUS-PPG measurement is excellent with no severe adverse events, indicating promising safety of this procedure. As reported by Choi et al., a major advantage of EUS-PPG is the ability to measure direct PPG as opposed to the IR guided transjugular technique that provides HVPG (IR-HVPG), an indirect measurement of portal pressure gradient (2). Therefore, EUS-PPG also offers the ability to generate useful data when evaluating patients with pre-hepatic or presinusoidal disease (2). Additionally, in contrast to IR-HVPG, the EUS-PPG measurement technique does not depend on ionizing radiation and intravenous contrast agents. It is worth considering that the threshold for CSPH (>10mmHg) is defined by the IR-HVPG technique, and it is yet to be confirmed whether this value can be used definitively for EUS-PPG. Currently, there are no direct head-to-head comparisons of EUS-HVP against IR-FHVP or EUS-PVP against IR-WHVP. However, EUS-PPG measurements have been found to correlate with both clinical evidence of cirrhosis and histologic evidence of liver fibrosis (2).

Liver biopsy with histopathologic examination is the gold standard for diagnosis of chronic liver disease and cirrhosis. Likewise, patient selection and consideration of other comorbid conditions for performing EUS-PPG and EUS-LB is critical. In patients with chronic liver disease of uncertain etiology, endoscopic evaluation with EUS provides not only the ability to screen for esophageal or gastric varices, but it also allows the simultaneous procedures of directly measuring PVP and performing EUS-guided elastography and EUS-LB (13). This is particularly important as it allows for a more comprehensive examination under a single exposure to anesthesia and simplifies the coordination of care of patients with chronic liver disease (8). In contrast, IR-HVPG does not allow for simultaneous endoscopic evaluation for esophageal or gastric varices. However, in high-risk patients, such as those with severe thrombocytopenia, coagulopathy, and/or large volume ascites, there are significant risks of complications of EUS-PPG and EUS-LB including bleeding and infection. In certain situations, IR-HVPG may be offered as an alternative option to indirectly measure PVP and provide a liver biopsy via transjugular approach. Nevertheless, EUS-PPG is favored over IR-HVPG in evaluation of patients with PH due to Budd Chiari syndrome/portal vein thrombosis as IR-HVPG is contraindicated in this setting due to risks of dislodgement of thrombi resulting in embolization. Presence of ascites is a relative contraindication to performing percutaneous LB, whereas both EUS-LB and transjugular LB can be considered, albeit with higher complication risks (7).

Despite the high rates of technical success and limited adverse effects, the EUS-PPG procedure itself is relatively new and is not widely available in centers across the country. Similar to newly developed medications or innovative techniques, with accumulation of data on efficacy and safety, increased utilization of the interventions is expected. Notably, one study reported death in a patient due to severe presentation of Budd Chiari syndrome and multiple co-morbidities. This event was unrelated to EUS-PPG measurement given that the procedure itself was successful without evidence of procedure related adverse events (11). An additional barrier to widespread use of a new technique is the requirement of new, expensive equipment to perform a given procedure. However, all studies that were included used the transgastric transhepatic approach with a compact manometer with a pressure transducer and a non-compressible tubing catheter, which are not major upgrades compared to the equipment of a standard endoscopy center. A specialized training in advanced endoscopy with special interest in endo-hepatology is required to perform EUS-PPG measurement. Lack of expertise is another barrier for widespread availability of this procedure in clinical practice. Finally, EUS-PPG measurement requires moderate sedation or general anesthesia, with the majority of studies using general anesthesia when performing simultaneous EUS-PPG and EUS-LB (8, 12). Previous literature has described variability in IR-HVPG measurements related to patients movements and cough, which can increase intra-abdominal pressure when patients are under inadequate sedation (14, 15). Currently, there are no available publications examining the influence of sedation exposure and EUS-PPG measurements (7, 14, 15). To date, a retrospective review comparing patients who underwent EUS-PPG with monitored anesthesia care (MAC) vs general anesthesia was performed at a single institution (16). The authors demonstrated that MAC was superior in terms of anesthesia time, but that both sedation methods had comparable safety and efficacy (16). EUS-PPG should be performed under MAC or general anesthesia given the benefits of sustained pressure recording. Inadequate conscious sedation may result in inaccurate PPG measurements. The limitations of this study are small sample size and lack of clinical trials given limited published data on this novel technique. In addition, only limited numbers of institutes are performing the EUS-PPG measurement procedure in the United States due to lack of expertise. Consequently, the data sets available for analysis were limited and not suitable for a meta-analysis. Secondly, of the studies that met eligibility for inclusion in the review, only two complete data sets that reported on the simultaneous EUS-PPG measurement and liver biopsy procedures were included. Although the results published by Hajifathalian et al. in 2022 did not demonstrate a statistically significant correlation between the fibrosis stage on histology and measured PPG, the authors acknowledged that their study lacked statistical power due to small sample size (n=24) (12). The subsequent work published by Choi et al. in 2022, including a data set of 64 patients, demonstrated that EUS-PPG >5 mmHg was significantly associated with fibrosis stage ≥ 3 on EUS-LB (2). The results from the present review emphasize the need for randomized controlled trials and multicentered prospective studies for assessing the external validity of the published data. Finally, none of the studies included in the review included data on long-term outcomes of patients who underwent EUS-guided PPG measurement with or without liver biopsy.

## Conclusion

The efficacy of EUS-PPG measurement is equivalent to IR-HVPG yet associated with less adverse events related to contrast media and exposure to ionizing radiation. The EUS-PPG measurement procedure offers the ability to perform a simultaneous EUS-LB, evaluation of esophageal or gastric varices and portal hypertensive gastropathy, preventing the need for a separate EGD. As the EUS-PPG procedure availability expands beyond only highly specialized endoscopy centers, further studies are needed to set patient selection criteria for candidacy of EUS-PPG measurement over IR-HVPG and to determine if there is a consistent correlation between EUS-PPG measurements and histologic staging of liver fibrosis on LB.

## Data availability statement

The original contributions presented in the study are included in the article/supplementary material. Further inquiries can be directed to the corresponding author.

## Author contributions

AM participated in project planning, data acquisition and review, manuscript writing, review, and editing. MY participated in project planning, data acquisition and review, manuscript review, and editing. GH participated in manuscript review, and editing. All authors contributed to the article and approved the submitted version.
